# FedSW-TSAD: SWGAN-Based Federated Time Series Anomaly Detection

**DOI:** 10.3390/s25134014

**Published:** 2025-06-27

**Authors:** Xiuxian Zhang, Hongwei Zhao, Weishan Zhang, Shaohua Cao, Haoyun Sun, Baoyu Zhang

**Affiliations:** 1 Qingdao Institute of Software, College of Computer Science and Technology, China University of Petroleum (East China), Qingdao 266580, China; 2 Shandong Data Open Innovative Application Laboratory, Qingdao 266580, China; 3 Shandong Key Laboratory of Intelligent Oil & Gas Industrial Software, Qingdao 266580, China

**Keywords:** anomaly detection, privacy protection, federated learning

## Abstract

As distributed sensing technologies evolve, the collection of time series data is becoming increasingly decentralized, which introduces serious challenges for both model training and data privacy protection. In response to this trend, federated time series anomaly detection enables collaborative analysis across distributed sensing nodes without exposing raw data. However, federated anomaly detection experiences issues with unstable training and poor generalization due to client heterogeneity and the limited expressiveness of single-path detection methods. To address these challenges, this study proposes FedSW-TSAD, a federated time series anomaly detection method based on the Sobolev–Wasserstein GAN (SWGAN). It leverages the Sobolev–Wasserstein constraint to stabilize adversarial training and combines discriminative signals from both reconstruction and prediction modules, thereby improving robustness against diverse anomalies. In addition, FedSW-TSAD adopts a differential privacy mechanism with L2-norm-constrained noise injection, ensuring privacy in model updates under the federated setting. The experimental results determined using four real-world sensor datasets demonstrate that FedSW-TSAD outperforms existing methods by an average of 14.37% in the F1-score while also enhancing gradient privacy under the differential privacy mechanism. This highlights the practical value of FedSW-TSAD for privacy-preserving anomaly detection in sensor-based monitoring systems such as industrial IoT, remote diagnostics, and predictive maintenance.

## 1. Introduction

With the rapid development of distributed sensing and edge computing, massive volumes of time series data are continuously collected and stored by various sensors [1]. These multivariate time series often exhibit complex inter-variable correlations and temporal structures, which can be leveraged by time series anomaly detection (TSAD) methods to identify abnormal points or segments that deviate from normal patterns. Accordingly, TSAD has become a core technique for monitoring dynamic systems across domains such as industrial process control [2,3], sensor-based healthcare monitoring [4], and critical infrastructure protection [5].

Most existing TSAD methods are designed for centralized settings and typically assume access to all training data on a single node [6]. However, in real-world deployments, time series data are often generated and stored locally by different nodes that form distributed sensor networks. Due to privacy concerns and bandwidth constraints, these nodes cannot upload raw data to a central server, making centralized TSAD approaches difficult to apply. This not only hinders the effective utilization of locally collected data but also limits the scalability and practicality of distributed sensor networks.

Facing these limitations, federated learning [7] (FL) offers a practical solution by enabling collaborative model training across distributed nodes, each treated as a federated client, without sharing raw data. In an FL framework, local models are trained independently on clients and periodically aggregated by a central server to form a global model. This paradigm has been successfully applied to privacy-sensitive domains, where it effectively balances collaboration and privacy [8]. Nonetheless, applying TSAD in federated environments is far from trivial, as it involves several non-trivial challenges that merit closer examination.

One major challenge in federated TSAD settings is the instability of model training caused by data heterogeneity and inconsistent local optimization [6]. Unsupervised methods are widely adopted for TSAD due to the scarcity of labeled anomalies, among which Generative Adversarial Networks (GANs) [9] have shown particular promise for modeling complex temporal patterns. However, despite their potential, GAN-based anomaly detection methods face significant stability issues, such as mode collapse and training failure, which are widely observed in empirical studies [10]. These issues can prevent the generator from effectively capturing the complex distribution of normal time series data. In a federated setting, this challenge is further exacerbated by client data heterogeneity, where local models may learn divergent data modes, leading to unstable global aggregation and degraded convergence behavior. These problems highlight the need for more stable generative architectures tailored to federated TSAD.

**Figure 1:**
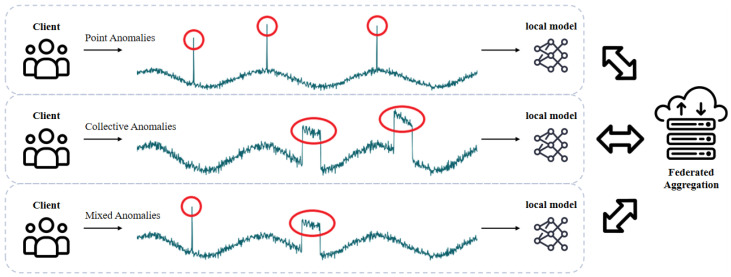
An illustration of heterogeneous anomaly types across clients in federated time series anomaly detection. Red circles highlight anomalous regions. Each client retains its local time series data and independently trains a local model, which contributes to the global model through federated aggregation.

Another key challenge lies in the diversity of anomaly types across clients, which limits the generalization ability of conventional detection paradigms. In unsupervised TSAD, reconstruction-based methods aim to identify anomalies by measuring reconstruction errors, which is typically effective for contextual or collective anomalies. In contrast, prediction-based methods forecast future values and flag deviations as anomalies, showing better sensitivity to point anomalies or abrupt changes [11]. As illustrated in Figure 1, federated clients often encounter heterogeneous anomaly types due to local sensor heterogeneity and diverse operational contexts. Consequently, single-path detection methods struggle to generalize across the federation. A hybrid scoring mechanism that integrates both reconstruction and prediction paths is needed to ensure robust performance under heterogeneous anomaly types across clients in distributed sensor networks.

Building on these observations, this study proposes FedSW-TSAD, a federated time series anomaly detection framework based on an improved Sobolev–Wasserstein GAN (SWGAN). First, to ensure stable training in federated settings, FedSW-TSAD introduces an enhanced SWGAN module, which replaces the Jensen–Shannon (JS) divergence used in standard GANs with the Sobolev–Wasserstein (SW) constraint [12]. The SWGAN module incorporates a Temporal Convolutional Network (TCN) into its generator to capture long-range dependencies and support the high-quality reconstruction of multivariate sequences. This integration enables robust and fine-grained modeling of multivariate time series, improving the detection performance in decentralized environments. Second, to address the heterogeneity of anomaly types across clients, FedSW-TSAD employs an additional prediction-based module. This module is co-trained with the SWGAN model to jointly optimize detection performance. During inference, anomaly scores from both modules are fused into a unified detection metric, leveraging the complementary strengths of reconstruction and prediction. Finally, to preserve privacy throughout the federated training process, an L2-norm-constrained noise-injection mechanism is applied to model updates. This mechanism enforces formal privacy guarantees while maintaining model utility. Together, these design choices result in stable training, robust detection across heterogeneous clients, and effective privacy protection. FedSW-TSAD further achieves significant performance gains over existing baselines, making it a practical and effective solution for federated anomaly detection in sensor-based monitoring systems.

The main contributions of this study are as follows:

- A novel framework named FedSW-TSAD is proposed, incorporating an improved Sobolev–Wasserstein GAN with a Temporal Convolutional Network. This design leads to more stable convergence and better anomaly detection performance in federated learning over distributed sensor networks.
- A hybrid scoring mechanism is developed, where reconstruction-based and prediction-based modules are jointly optimized to leverage their complementary strengths. This approach improves robustness in complex distributed environments with diverse anomaly types and client behaviors.
- Comprehensive experiments are conducted on four real-world sensor datasets. FedSW-TSAD consistently outperforms both centralized and federated baselines, achieving average F1-score improvements of 4.27% and 14.37% over the strongest centralized and federated baselines, respectively. Furthermore, a case study demonstrates that the proposed differential privacy mechanism reduces gradient leakage risk.

The remainder of this study is structured as follows. Section 2 reviews related work on time-series anomaly detection and federated learning. Section 3.1 introduces the SW-TSAD model, which serves as the centralized backbone combining Sobolev–Wasserstein-based adversarial reconstruction mechanisms and hybrid anomaly scoring. Section 3.2 extends the framework to the federated setting, proposing FedSW-TSAD with privacy-preserving and robustness-enhancing techniques. Section 4 presents the experimental setup, baseline comparisons, and quantitative results. Section 5 offers a comprehensive analysis including ablation, efficiency, robustness, hyperparameter sensitivity, and the impact of privacy mechanisms. Finally, Section 6 concludes the paper and outlines future directions.

## 2. Related Work

### 2.1. Time Series Anomaly Detection

Time series anomaly detection is a reliable solution for maintaining modern systems’ safety and has long been a prominent research focus. Due to the scarcity of labeled anomalies, unsupervised learning is the dominant paradigm in most practical TSAD applications. Under this paradigm, existing TSAD methods can be broadly categorized into classical statistical approaches, prediction-based techniques, and reconstruction-based techniques.

**Classical statistical methods** have historically served as foundational tools in unsupervised TSAD. One representative approach is the Local Outlier Factor (LOF) [13], which detects anomalies by measuring the local data density. The Isolation Forest (IF) [14], in contrast, detects anomalies by randomly partitioning data points and measuring the ease of isolation. A notable early attempt to model temporal dependencies is the Hidden Markov Model (HMM) [15], which assumes fixed-order Markovian dynamics and predefined transition structures. Other commonly used techniques include PCA [16], KNN [17], and one-class SVM (OC-SVM) [18]. While computationally lightweight and interpretable, these methods often rely on strong distributional assumptions and struggle to capture the nonlinear or temporal dependencies in multivariate time series. As a result, they are often outperformed by deep learning methods in complex real-world scenarios.

**Prediction-based methods** identify anomalies by first forecasting future values, then measuring the deviation between predicted and observed data points. In practice, researchers typically train a prediction model on normal time series to learn temporal patterns, and prediction errors are used as anomaly scores during inference [19,20,21]. Accordingly, several deep models have shown promising results in different application scenarios. Neural architectures such as LSTM-based models [22,23] achieve strong performance in telemetry systems. In addition, hybrid frameworks combining VAE and GBDT [24] have demonstrated utility in smart grid monitoring. Prediction-based methods are particularly effective in capturing abrupt deviations, making them well-suited for detecting point anomalies. However, their reliance on short-term forecasting limits their ability to handle contextual and collective anomalies with complex temporal structures. Beyond anomaly detection, it is worth noting that time series prediction itself has been widely studied in various application scenarios. For example, models based on recurrent neural networks and fully connected architectures have demonstrated strong capabilities in COVID-19 trend forecasting [25] and high-speed train vibration prediction [26], highlighting the importance of predictive modeling for complex temporal systems.

**Reconstruction-based methods** detect anomalies by learning low-dimensional representations of normal patterns and identifying deviations in reconstructions. These models reconstruct inputs from compressed representations and assume anomalies cannot be accurately recovered due to their rarity and unpredictability. Approaches including LSTM-Autoencoders [27,28], Variational Autoencoders (VAEs) [29,30], and DAGMM [31] have demonstrated effectiveness in modeling complex multivariate time series. By modeling long-range temporal dependencies, reconstruction-based methods are positioned well to identify contextual and collective anomalies. Nevertheless, these methods often result in false negatives and unstable training behavior.

Among reconstruction-based methods, Generative Adversarial Networks have attracted considerable attention due to their powerful generative capabilities and potential to model complex temporal dependencies. Representative methods such as MAD-GAN [32] and TadGAN [33] leverage adversarial training to improve reconstruction fidelity and capture nonlinear dynamics in multivariate TSAD tasks. Despite these advantages, GAN-based models also suffer from notorious training issues like mode collapse and gradient vanishing. Such instability tends to be exacerbated in federated environments, where data heterogeneity and inconsistent local updates undermine convergence. Consequently, existing approaches still fail to provide a robust TSAD framework that combines the strengths of reconstruction and prediction while ensuring stable training under distributed sensor networks.

### 2.2. Federated Learning

Federated Learning (FL) [7], an innovative distributed machine learning framework, facilitates collaborative model training without requiring direct access to raw data. It has gained increasing attention in privacy-sensitive domains, including IoT and healthcare, where time series data are naturally distributed across edge devices. For instance, DIoT [34] demonstrates the effectiveness of FL-based anomaly detection in device communication monitoring, while [35] applies FL to cross-institutional healthcare data to detect abnormal patterns early.

Recent studies have also systematically investigated the integration of federated learning with TSAD. These studies explore the challenges and solutions for time series anomaly detection in federated settings, which is most relevant to our work. FedTADBench [36] provides a systematic benchmark, evaluating the performance of mainstream TSAD algorithms (e.g., LSTM-AE, USAD, and GDN) under federated settings with varying client partitions and aggregation strategies. The benchmark primarily reports AUC-ROC and AUC-PR as performance metrics across different scenarios. PeFAD [6] improves communication efficiency by employing pre-trained language models (PLMs) as the local model backbone, a parameter-efficient training scheme, and knowledge distillation on a synthetic shared dataset. Its performance is evaluated in terms of its F1-score, detection accuracy, and communication cost. FedAnomaly [37] adopts a variational autoencoder with convolutional and recurrent encoders (ConvGRU) to model spatiotemporal dependencies at the edge and evaluates its performance in terms of its F1-score, detection latency, and ability to detect contiguous anomaly segments.

Although these methods demonstrate promising results, most rely solely on either reconstruction or prediction-based strategies, limiting their generalization under diverse anomaly types. Furthermore, while some studies have explored the combination of federated learning with differential privacy in other domains, such as intrusion detection systems [38], similar efforts remain rare in federated TSAD. Generative instability also remains underexplored, especially in federated environments, where non-IID data and inconsistent local updates often exacerbate convergence issues. To address these gaps, FedSW-TSAD was created: a framework designed to stabilize adversarial training, integrate complementary reconstruction and prediction signals, and incorporate differential privacy mechanisms to improve robustness and preserve privacy across FL clients.

## 3. Materials and Methods

This section presents the proposed frameworks for time series anomaly detection. It first introduces SW-TSAD, a centralized model that integrates adversarial reconstruction and temporal prediction. Building upon this foundation, FedSW-TSAD is developed to enable privacy-preserving federated training across distributed sensor networks.

### 3.1. SWGAN-Based Time Series Anomaly Detection

SW-TSAD serves as the local modeling framework of FedSW-TSAD, combining adversarial reconstruction and temporal prediction to enable robust time series anomaly detection. It begins by formalizing the problem of multivariate time series anomaly detection, followed by a detailed presentation of the proposed model, which integrates a Sobolev-Wasserstein GAN for generative reconstruction and a parallel LSTM-based predictor for temporal forecasting. The joint training and hybrid scoring mechanism are further elaborated, along with the complete algorithmic workflow.

#### 3.1.1. Problem Description

In multivariate time series anomaly detection, the input data is typically collected by a network of sensors. Each sensor continuously records measurements over time, forming a multivariate time series. Formally, let $x=[x_{1},x_{2},\ldots ,x_{T}]$ denote a multivariate time series of length *T*, where each observation $x_{t}\in R^{M}$ represents measurements from *M* sensors (or features) at time step *t*. Thus, the entire time series x has a matrix form of size $R^{T\times M}$. To facilitate training, x is segmented into *N* fixed-length subsequences of size *S*, using a sliding window with stride *d*. Here, the stride *d* determines the step size between two adjacent windows, and $N=\left\lfloor \frac{T-S}{d}\right\rfloor +1$. This segmentation yields a training tensor $X\in R^{N\times S\times M}$, where each subsequence $x_{i,1:S}\in R^{S\times M}$ is a contiguous temporal window of x.

Based on the segmented input, the TSAD model in this paper is designed to jointly optimize three components: a predictor *P*, a discriminator *D*, and a generator *G*, with corresponding parameters Θ=(η,ω,θ). In the training phase, the objective is to minimize the average loss across all *N* input subsequences:

(1) $$\Theta^{*}=\arg\min_{\Theta }\frac{1}{N}\sum_{i=1}^{N}L\left(\Theta ;x_{i,1:S}\right)$$

where $L(\Theta ;x_{i,1:S})$ denotes the loss function for the *i*-th subsequence.

During inference, the trained model with optimal parameters $\Theta^{*}=\left(\eta^{*},\omega^{*},\theta^{*}\right)$ is applied to a set of subsequences $X^{\prime }=\langle x_{1,1:S}^{\prime },x_{2,1:S}^{\prime },\ldots ,x_{T^{\prime },1:S}^{\prime }\rangle$ extracted from the test time series $x^{\prime }$. For each time step *t*, an anomaly score is computed as follows:

(2) $$AD_{score}^{\left(t\right)}=\alpha R_{score}^{\left(t\right)}\left(x_{t,1:S}^{\prime };\theta^{*}\right)+\beta D_{score}^{\left(t\right)}\left(x_{t,1:S}^{\prime };\omega^{*}\right)+\gamma P_{score}^{\left(t\right)}\left(x_{t,1:S}^{\prime };\eta^{*}\right)$$

where $R_{score}$, $D_{score}$, and $P_{score}$ denote the reconstruction, discrimination, and prediction scores, with α, β, and γ as their respective weights. A higher $AD_{score}^{\left(t\right)}$ indicates a higher likelihood that the observation at time step *t* is anomalous.

The final output of the TSAD model is a binary indicator sequence $\hat{y}\in {\left\{0,1\right\}}^{T}$, where ${\hat{y}}^{\left(t\right)}=1$ denotes an anomaly at time *t*. This is achieved by labeling time steps as anomalies if their anomaly scores $AD_{score}^{\left(t\right)}$ exceed a predefined threshold.

#### 3.1.2. Overall Architecture

**Figure 2:**
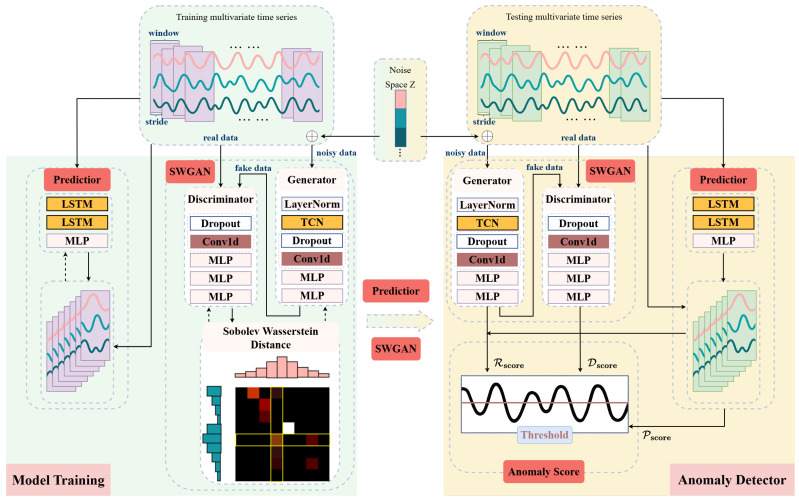
The architecture of the proposed SW-TSAD framework. SW-TSAD stands for the time series anomaly detection using Sobolev-Wasserstein GAN (SWGAN).

Figure 2 illustrates the comprehensive architecture of the proposed SW-TSAD model, which serves as the core local anomaly detection component within the broader FedSW-TSAD framework. The input time series waveform is first processed through a fixed-size sliding window mechanism, generating a set of overlapping subsequences. Each subsequence, representing a temporal segment of the raw data, then serves as the input to the SW-TSAD model. The model comprises two primary, jointly optimized modules: a Sobolev–Wasserstein Generative Adversarial Network (SWGAN) module and a prediction module. The SWGAN module trains a generator and a discriminator using real normal data, generator-produced (fake) data, and noise-corrupted data to learn structural and distributional properties of normal subsequences by minimizing the Sobolev-Wasserstein distance between them. This design improves the stability of adversarial training and enhances the model’s sensitivity to subtle anomalies. Concurrently, the prediction module utilizes a Temporal Convolutional Network and Long Short-Term Memory (LSTM) layers to capture long-range temporal dependencies and forecast future time series behaviors. Through this joint optimization, the model learns to encode both temporal dependencies and distributional regularities of normal sequences, capturing complementary aspects of normal time series behavior—namely structural reconstruction, adversarial discrimination, and temporal forecasting. During inference, the trained model assigns an anomaly score to each input segment based on deviations from these learned normal patterns. Specifically, the final anomaly score is robustly obtained by combining distinct signals from both the SWGAN (reflecting reconstruction and discrimination errors) and the prediction modules (quantifying forecasting errors). This fusion of complementary anomaly signals significantly enhances the model’s capacity to detect various anomalies with diverse temporal and structural characteristics. To facilitate a deeper understanding of SW-TSAD, the structure and specific roles of each module are detailed in the subsequent sections.

The SWGAN module comprises a generator and a discriminator. The generator performs conditional reconstruction using input subsequences perturbed with Gaussian noise and mapping them onto the original data manifold. Aided by a Temporal Convolutional Network, it captures long-range temporal dependencies to enhance reconstruction quality. The discriminator, on the other hand, evaluates the realism of generated samples to guide the generator via adversarial feedback. Unlike traditional GANs that rely on Jensen–Shannon divergence and often suffer from instability such as mode collapse or vanishing gradients, this design replaces JS divergence with the SW constraint to enforce a smoother Lipschitz constraint and promote more stable training.

The prediction module is implemented using a two-layer LSTM followed by a linear output layer. It learns to forecast future values based on past observations, thereby capturing sequential dependencies that may not be explicitly modeled by the generator.

To produce the final anomaly score, SW-TSAD combines the outputs from all three branches: the reconstruction score $R_{score}$ from the generator, the discrimination score $D_{score}$ from the discriminator, and the prediction score $P_{score}$ from the predictor. This hybrid scoring strategy allows the model to leverage both reconstruction fidelity and temporal consistency. As shown in Figure 2, test data are processed in a sliding-window fashion, where each subsequence is evaluated by all three branches. The final anomaly score is computed through a weighted combination of the three outputs, which is further detailed in Section 3.1.4.

#### 3.1.3. SWGAN Module

Adversarial learning in GANs is known to suffer from instability issues, such as mode collapse, vanishing gradients, and poor convergence. These problems are particularly critical for time series anomaly detection, where the generator must accurately learn the distribution of normal time series patterns. If the generator fails to model this distribution, it produces biased or incomplete reconstructions, causing the reconstruction errors to lose their discriminative power. As a result, both false alarms and missed anomalies may occur, degrading the model’s reliability. The fundamental cause of these instability issues lies in using the Jensen–Shannon divergence as the optimization objective in standard GANs. To address this, we adopt the Sobolev–Wasserstein constraint, which replaces the JS divergence and provides a more stable and theoretically grounded alternative. The original formulation of the SWGAN is defined as follows:

(3) $$\begin{array}{cc} \min_{G}\max_{D}L_{S}\left(D_{w},G_{\theta }\right) & =E_{x∼P_{r}}D_{w}\left(x\right)-E_{z∼P_{z}}D_{w}\left(G_{\theta }\left(z\right)\right), \end{array}$$

with the constraint that

(4) $$E_{x∼\mu^{x_{i},x_{j}}}{∥\nabla_{x}D_{w}\left(x\right)∥}^{2}\leq 1,\forall x_{i}∼P_{r},\forall x_{j}∼P_{g}$$

where $G_{\theta }$ is the generator parameterized by θ, $D_{w}$ is the discriminator parameterized by *w*, $P_{r}$ is the real data distribution, $P_{g}$ is the generator-induced distribution, $P_{z}$ is the distribution of latent noise vectors *z* used as inputs for the generator (typically a Gaussian or uniform distribution), and $\mu^{x_{i},x_{j}}$ denotes the uniform interpolation distribution between real subsequences $x_{i}∼P_{r}$ and generated subsequences $x_{j}∼P_{g}$. The constraint enforces smooth discriminator behavior across the interpolated input space, helping stabilize adversarial training and improving generalization.

Although the original SWGAN enforces this constraint via Lagrangian multipliers and slack variables [12], such formulations increase optimization complexity and are sensitive to hyperparameters. To simplify training and improve stability, SW-TSAD retains the core objective of SWGAN but replaces the constraint mechanism with a gradient penalty, which softly enforces the Lipschitz condition by adding a regularization term to the discriminator loss. This formulation eliminates the need for auxiliary variables and enables more stable adversarial optimization. Based on the reformulated objective, the implementation details of the SWGAN module are introduced below.

Given a time series segmented into *N* fixed-length subsequences ${\left\{x_{i,1:S}\right\}}_{i=1}^{N}$, which collectively form the training set, the generator G(·;θ) reconstructs the values for time steps in $\left[1,t_{0}\right]$, where $t_{0}$ denotes the reconstruction horizon. A noise term $z\in R^{t_{0}\times M}$, sampled from a standard normal distribution N(0,1), is added to the target segment to introduce stochasticity into the generative process. As a result, the reconstructed segment is given by ${\hat{x}}_{i,1:t_{0}}=G\left(x_{i,1:t_{0}}+z;\theta \right)$. The discriminator model D(·;ω) then validates the closeness between the output value of G(·;θ) and the true value of the target range. The objective function for the discriminator is given by

(5) $$\begin{array}{cc} L_{D} & =\frac{1}{N}\sum_{i=1}^{N}D\left({\hat{x}}_{i,1:t_{0}};\omega \right)-\frac{1}{N}\sum_{i=1}^{N}D\left(x_{i,1:t_{0}};\omega \right)+\lambda \frac{1}{N}\sum_{i=1}^{N}\left({\left({∥\nabla_{{\bar{x}}_{i}}D\left({\bar{x}}_{i};\omega \right)∥}_{2}-1\right)}^{2}\right) \end{array}$$

where $\bar{x_{i}}=\epsilon \cdot x_{i,1:t_{0}}+\left(1-\epsilon \right)\cdot {\hat{x}}_{i,1:t_{0}}$ and ϵ∼U[0,1]. Here, λ is a regularization coefficient that controls the strength of the Sobolev gradient penalty.

The objective function of the generator is

(6) $$L_{G}=-\frac{1}{N}\sum_{i=1}^{N}D\left({\hat{x}}_{i,1:t_{0}};\omega \right)$$

This adversarial training process proceeds by alternately updating the discriminator and generator. The discriminator learns to maximize its ability to distinguish real from generated data, while the generator is optimized to fool the discriminator by generating reconstructions that align closely with the structural characteristics of normal data. To further support this objective, a Temporal Convolutional Network is incorporated into the generator to improve its ability to model complex temporal patterns and capture long-range dependencies.

**Figure 3:**
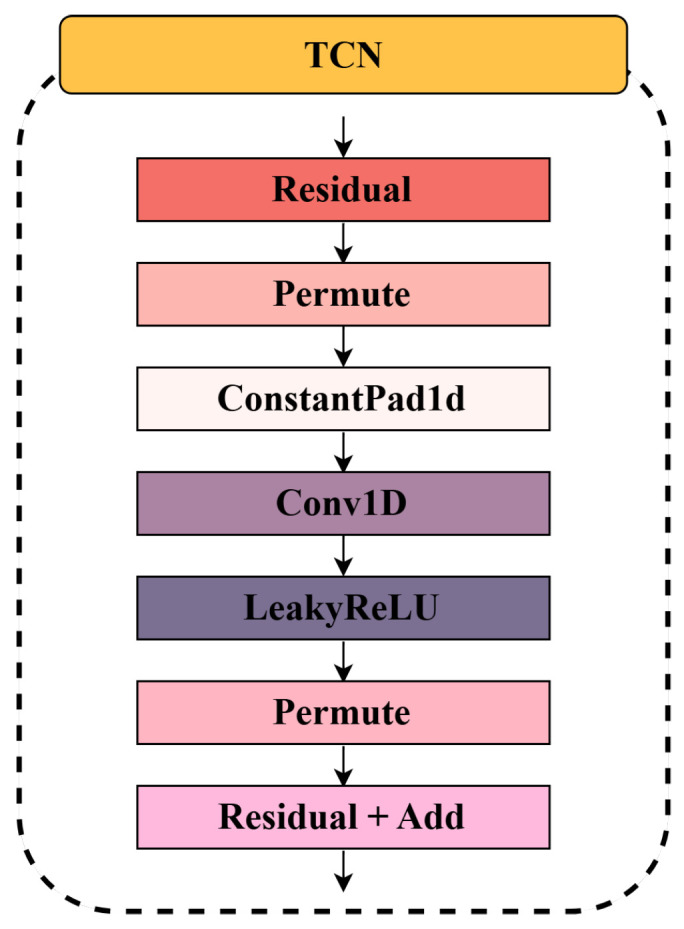
The architecture of the Temporal Convolutional Network (TCN) in the SWGAN module.

As shown in Figure 3, the Temporal Convolutional Network in SW-TSAD is designed to efficiently capture temporal dependencies while maintaining stability and computational efficiency. The architecture is structured as follows:

1. Residual Block Design: TCN employs residual connections to facilitate stable gradient propagation and accelerate convergence. Each residual block consists of multiple convolutional layers interleaved with non-linear activations, enabling effective feature extraction while preserving historical information.
2. Temporal Processing via Convolutional Layers: The network utilizes 1D convolutional layers (Conv1D) to model sequential dependencies. To expand the receptive field without disrupting temporal alignment, a constant padding (ConstantPad1d) operation is applied before convolution.
3. Activation and Transformation: Each convolutional layer is followed by a LeakyReLU activation function, which introduces non-linearity while mitigating vanishing gradient issues. Permutation operations (Permute) are applied before and after convolution to ensure proper dimensional alignment for temporal sequence processing.
4. Residual Connections and Feature Fusion: The output from the convolutional layers is merged with the original input via an addition operation (Residual + Add), preserving low-level information and enhancing gradient flow across layers. This design mitigates degradation issues in deep networks and promotes feature reuse.

Overall, the TCN module enables the generator to extract multi-scale temporal features from noisy or corrupted input segments, contributing to the robustness of sequence reconstruction in the adversarial setting. The SWGAN module and its integrated TCN structure together form a robust generative backbone that effectively captures the temporal and structural regularities of normal time series data, serving as a foundation for subsequent anomaly scoring.

#### 3.1.4. Prediction Module and Anomaly Scoring

In addition to generative modeling, SW-TSAD incorporates a dedicated prediction module to capture temporal dependencies through forecasting. While the SWGAN module emphasizes distributional reconstruction, the prediction module provides an orthogonal perspective by learning temporal continuity, enabling complementary scoring for anomaly detection.

As visualized in Figure 2, the prediction module consists of two LSTM layers followed by a linear, fully connected layer. The predictor *P* takes multivariate time series data of a given window length as input and outputs predicted time series data for the target window length. Additionally, *P* is trained in parallel with the SWGAN module. During the testing phase, the prediction error is computed and incorporated into anomaly scoring.

Given an input time series $x_{i,1:S},i=1,\ldots ,N$, the prediction model P(·;η) with parameter η predicts the values for time steps in $\left[t_{0}+1,t_{0}+\tau \right]$ conditioning on time steps in $\left[1,t_{0}\right]$. τ is the number of time steps P(·;η) is trained to predict, where $t_{0}+\tau =S$. In contrast to the generator, which reconstructs the target segment based on noisy inputs, the predictor learns to forecast future observations from past contexts. That is, ${\tilde{x}}_{i,t_{0}+1:S}=P\left(x_{i,1:t_{0}};\eta \right).$ The time ranges $\left[1,t_{0}\right]$ and $\left[t_{0}+1,S\right]$ are referred to as the conditioning range and target range, respectively. The predictor is trained using the general Mean Squared Error (MSE) loss, defined as

(7) $$L_{P}=\frac{1}{N}\sum_{i=1}^{N}{\left\|x_{i,t_{0}+1:S}-{\tilde{x}}_{i,t_{0}+1:S}\right\|}^{2}$$

With the training objectives of the SWGAN and the prediction module clearly defined, both components are optimized in parallel during model training. This joint learning enables the model to capture the complementary structural and temporal features of normal time series patterns.

Once the training is complete, the optimized model parameters $\Theta^{*}=\left(\eta^{*},\omega^{*},\theta^{*}\right)$ are deployed to perform anomaly detection on test sequences $x^{\prime }$. Inspired by the DR score in MAD-GAN [32], SW-TSAD defines the anomaly score ($AD_{score}$) as a combination of the generator’s reconstruction error ($R_{score}$), the discriminator’s discrimination error ($D_{score}$), and the predictor’s prediction error ($P_{score}$). For each time step *t*, the anomaly score is computed as follows:

(8) $$R_{score}^{\left(t\right)}=\frac{1}{\tau }{\;\|\;x_{t,t_{0}+1:S}^{\prime }-G\left(x_{t,t_{0}+1:S}^{\prime }+z;\theta^{*}\right)\;\|\;}^{2}$$

(9) $$D_{score}^{\left(t\right)}=-D\left(x_{t,t_{0}+1:S}^{\prime };\omega^{*}\right)+1$$

(10) $$P_{score}^{\left(t\right)}=\frac{1}{\tau }{\;\|\;x_{t,t_{0}+1:S}^{\prime }-P\left(x_{t,1:t_{0}}^{\prime };\eta^{*}\right)\;\|\;}^{2}$$

(11) $$AD_{score}^{\left(t\right)}=\alpha R_{score}^{\left(t\right)}+\beta D_{score}^{\left(t\right)}+\gamma P_{score}^{\left(t\right)}$$

where α, β, and γ are weight parameters satisfying α+β+γ=1, used to balance $R^{\left(t\right)}score$, $D^{\left(t\right)}score$, and $P_{score}^{\left(t\right)}$. These weights can be selected empirically based on validation performance. Anomaly scores are used to detect anomalous points based on a predefined threshold during the anomaly detection phase. The threshold can be set using various methods, and if a point’s anomaly score exceeds the threshold, it is classified as anomalous.

The introduction of the prediction module, along with the unified anomaly scoring mechanism, allows SW-TSAD to jointly exploit reconstruction, discrimination, and forecasting errors. This design enables the model to better capture both structural and temporal anomalies, ultimately enhancing detection performance across diverse time series patterns.

#### 3.1.5. SW-TSAD Workflow

To consolidate the design of SW-TSAD, this section summarizes the complete training workflow, which jointly optimizes the generative and predictive components. As detailed in Algorithm 1, the model operates in a hybrid optimization loop. In each training round, the predictor *P* and discriminator *D* are first updated using their respective loss functions (Equations (5) and (7)). These two components provide complementary learning signals—*P* captures temporal continuity via forecasting, while *D* focuses on structural regularity by distinguishing between real and generated sequences.
**Algorithm 1** SWGAN-based Time Series Anomaly Detection Strategy.  1:**Input:** training set X; testing set $X^{\prime }$; time window length *S*; conditional length $t_{0}$; target length τ; batch size *m*; gradient penalty coefficient λ; weight parameters (α,β,γ); discriminator update steps $n_{\mathrm{critic}}$; Adam optimizer parameters $\left(\phi ,\psi_{1},\psi_{2}\right)$; initialized parameters Θ=(η,ω,θ).  2:**Output:** Anomaly score $AD_{score}$.  3:**//*****Training Phase***  4:**while** not converged **do**  5:    **for** t=1 to $n_{\mathrm{critic}}$ **do**  6:        **for** i=1 to *m* **do**  7:           Sample real subsequence $x_{i,1:S}∼X$;  8:           Predict: ${\tilde{x}}_{t_{0}+1:t_{0}+\tau }=P\left(x_{i,1:t_{0}};\eta \right)$;  9:           Sample noise subsequence z∼N(0,1);10:           Reconstruct: ${\hat{x}}_{i,1:t_{0}}=G\left(x_{i,1:t_{0}}+z;\theta \right)$;11:           Compute discriminator loss $L_{D}^{\left(i\right)}$ and predictor loss $L_{P}^{\left(i\right)}$ using Equations (5) and (7);12:        **end for**13:        Compute gradients: $\nabla_{\eta }L_{P}=\frac{1}{m}\sum_{i=1}^{m}\nabla_{\eta }L_{P}^{\left(i\right)}$, $\nabla_{\omega }L_{D}=\frac{1}{m}\sum_{i=1}^{m}\nabla_{\omega }L_{D}^{\left(i\right)}$;14:        Update predictor: $\eta \leftarrow \mathrm{Adam}(\eta ,\nabla_{\eta }L_{P},\phi )$;15:        Update discriminator: $\omega \leftarrow \mathrm{Adam}(\omega ,\nabla_{\omega }L_{D},\phi ,\psi_{1},\psi_{2})$;16:    **end for**17:    Compute generator loss $L_{G}$ using Equation (6);18:    Compute gradients: $\nabla_{\theta }L_{G}=\frac{1}{m}\sum_{i=1}^{m}\nabla_{\theta }L_{G}^{\left(i\right)}$;19:    Update generator: $\theta \leftarrow \mathrm{Adam}(\theta ,\nabla_{\theta }L_{G},\phi ,\psi_{1},\psi_{2})$;20:**end while**21:Obtain the converged model $\Theta^{*}=\left(\eta^{*},\omega^{*},\theta^{*}\right)$;22:**//*****Inference Phase***23:**for** each test subsequence $x_{t,1:S}^{\prime }\in X^{\prime }$ **do**24:    Predict: ${\tilde{x}}_{t,t_{0}+1:S}^{\prime }=P\left(x_{t,1:t_{0}}^{\prime };\eta^{*}\right)$;25:    Sample noise subsequence z∼N(0,1);26:    Reconstruct: ${\hat{x}}_{t,t_{0}+1:S}^{\prime }=G\left(x_{t,t_{0}+1:S}^{\prime }+z;\theta^{*}\right)$;27:    Calculate anomaly scores $AD_{score}^{\left(t\right)}$ using Equations (8)–(11);28:**end for**29:**return** $AD_{score}$

Subsequently, the generator *G* is updated by minimizing the loss in Equation (6), encouraging the generation of realistic target segments that align with normal sequence dynamics. To improve adversarial stability, the discriminator is updated multiple times per generator iteration (denoted as $n_{\mathrm{critic}}$) while following the training scheme of WGAN-GP [39]. All components are optimized using the Adam optimizer. This coordinated learning strategy enables the model to progressively learn fine-grained temporal and structural features, laying the foundation for robust anomaly detection during inference.

After training converges, the optimized model parameters $\Theta^{*}=\left(\eta^{*},\omega^{*},\theta^{*}\right)$ are used to perform anomaly detection on unseen test sequences. For each test window, the predictor, generator, and discriminator, respectively, yield forecasting, reconstruction, and discrimination errors, which are fused into an anomaly score defined in Equations (8)–(11). This scoring mechanism helps accurately identify abnormal time points based on deviations from learned temporal and structural patterns.

### 3.2. Federated Time Series Anomaly Detection

In real-world scenarios, time series data are often distributed across multiple locations with strict privacy constraints, preventing the direct deployment of the previously introduced SW-TSAD. Thus, this section proposes FedSW-TSAD, which maintains the advantages of adversarial generation and temporal prediction while incorporating differential privacy mechanisms for secure and efficient collaboration.

#### 3.2.1. Scenario and Architecture

FedSW-TSAD operates in a federated sensing setting where each client holds private multivariate time series data. To preserve privacy, only model updates are exchanged, enabling the collaborative construction of a global anomaly detector without sharing raw data. Formally, let a federation consist of *K* clients. Each client *k* owns a local dataset $D_{k}=x^{k}$, where $x^{k}\in R^{T_{k}\times M}$ and $T_{k}$ denote the sequence length. The total number of time steps across all clients is given by $T_{\mathrm{total}}=\sum_{k=1}^{K}T_{k}$. Global anomaly detection is achieved by minimizing a weighted sum of client-specific objectives:

(12) $$\Theta_{g}^{*}=\arg\min_{\Theta_{g}}\sum_{k=1}^{K}\frac{T_{k}}{T_{\mathrm{total}}}L_{k}\left(\Theta_{g};D_{k}\right)$$

where $\Theta_{g}=\left(\eta_{g},\omega_{g},\theta_{g}\right)$ denotes the global model parameters extended from Algorithm 1, and $L_{k}$ is the loss function computed on client *k*.

**Figure 4:**
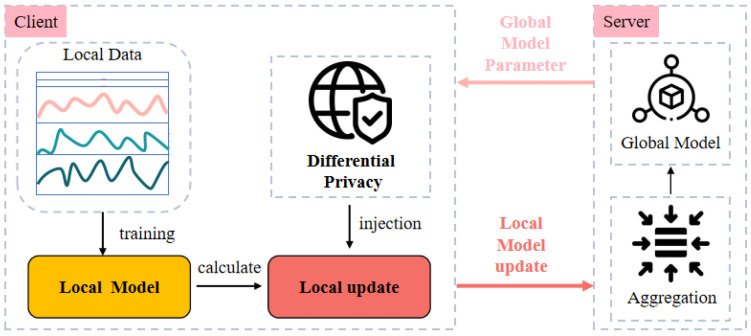
The architecture of the proposed FedSW-TSAD framework. FedSW-TSAD stands for federated time series anomaly detection using Sobolev–Wasserstein GAN.

The architecture of FedSW-TSAD is illustrated in Figure 4, which highlights the client-server interaction and privacy-preserving communication pipeline. Each communication round consists of two main steps: local update and global aggregation. In the local update phase, client *k* performs several training steps on its private dataset $D_{k}$, resulting in updated parameters $\Theta_{k}$ and a corresponding model update $\Delta_{\Theta_{k}}$. To protect local information, each client applies L2-norm constrained differential privacy noise to its local update $\Delta_{\Theta_{k}}$ before transmission:

(13) $${\tilde{\Delta }}_{\Theta_{k}}=P\left(\Delta_{\Theta_{k}}\right)+N\left(0,\sigma^{2}I\right)$$

where P(·) denotes the reprocessing operator, and σ controls the noise magnitude.

In the global aggregation phase, the central server aggregates noisy updates using weighted averaging:

(14) $$\Delta_{\Theta_{g}}^{\left(r\right)}=\sum_{k=1}^{K}\frac{T_{k}}{T_{\mathrm{total}}}{\tilde{\Delta }}_{\Theta_{k}}^{\left(r\right)}$$

where *r* denotes the communication round.

The global model is updated accordingly and broadcast to all clients for the next round. This iterative process allows the global model to gradually integrate knowledge from heterogeneous client distributions while rigorously preserving local privacy. By decoupling data access from training, FedSW-TSAD supports secure and scalable anomaly detection across decentralized time series sources.

#### 3.2.2. Regularization and Differential Privacy Protection

To protect individual-level information in client updates, FedSW-TSAD incorporates (ε,δ)-differential privacy via the standard Gaussian mechanism. Each client performs gradient clipping to limit update sensitivity, followed by noise injection calibrated to a desired privacy budget. This ensures that the global model cannot infer specific sample-level information from local updates. In addition, to address the instability caused by heterogeneous local datasets—which may lead to noisy or overfitted updates—FedSW-TSAD applies L2-norm regularization prior to privacy operations. This results in a two-fold mechanism: regularization stabilizes local learning, while privacy-preserving perturbation guarantees data protection.

**L2-norm Regularization.** As a first step, an L2-norm regularization is applied to suppress excessive dependence on local updates and enforce smoother gradient trajectories. Let $\Delta_{\Theta }$ denote the local parameter update after client-side training. The regularization objective is defined as the expected L2-norm of the update:

(15) $$R_{\mathrm{norm}}=\lambda_{r}\cdot E[∥\Delta_{\Theta }{∥}_{2}]$$

where $\lambda_{r}$ is a regularization strength coefficient.

Next, the local update is adjusted via gradient descent on this regularizer:

(16) $$\Delta_{\Theta }\leftarrow \Delta_{\Theta }-\nabla_{\Theta }R_{\mathrm{norm}}$$

The adjusted update $\Delta_{\Theta }$ then serves as the input for subsequent privacy-preserving operations, such as gradient clipping and Gaussian noise injection.

**Clipping and Noise Injection.** To satisfy the sensitivity constraints required by differential privacy, each client applies L2 clipping with a dynamic threshold *C*:

(17) $$\Delta_{\Theta }\leftarrow \frac{\Delta_{\Theta }}{\max(1,||\Delta_{\Theta }{\left|\right|}_{2}/C)},\;C=\frac{1}{\sqrt{d_{\mathrm{in}}}}$$

where $d_{\mathrm{in}}$ is the input dimensionality. This bounds the update norm by *C* and limits the maximum possible influence of any individual sample. Finally, Gaussian noise is added to achieve (ε,δ)-differential privacy:

(18) $$\varepsilon ∼N\left(0,\sigma^{2}I\right),\;\sigma =\frac{C\sqrt{2\ln(1.25/\delta )}}{\varepsilon }$$

For convenience, the regularization and clipping can be encapsulated by a unified operator P(·):

(19) $$P\left(\Delta_{\Theta }\right)=\mathrm{Clip}\left(\Delta_{\Theta }-\nabla_{\Theta }R_{\mathrm{norm}}\right)$$

Thus, the final privacy-preserving update transmitted to the server becomes

(20) $${\tilde{\Delta }}_{\Theta }=P\left(\Delta_{\Theta }\right)+N\left(0,\sigma^{2}I\right)$$

Through the combination of Sobolev regularization, sensitivity-aware clipping, and calibrated noise injection, FedSW-TSAD enforces robust and privacy-preserving parameter updates, ensuring that the global model is both generalizable and secure against gradient leakage.

#### 3.2.3. FedSW-TSAD Workflow

To complete the design of FedSW-TSAD, this section presents the federated training workflow, which extends the optimization strategy of SW-TSAD into a privacy-preserving collaborative setting. As detailed in Algorithm 2, the framework adopts a parameter-isolated architecture where raw data remain local and only differentially private model updates are exchanged between clients and the central server.
**Algorithm 2** FedSW-TSAD: Federated SWGAN-based Time Series Anomaly Detection.  1:**Input:** time series dataset $D={\left\{D_{k}\right\}}_{k=1}^{K}$; testing set $X^{\prime }$; initial global parameters $\Theta_{g}^{\left(1\right)}=\left(\eta_{g}^{\left(1\right)},\omega_{g}^{\left(1\right)},\theta_{g}^{\left(1\right)}\right)$; communication rounds $R_{\max}$; local training epochs *E*; DP budget (ϵ,δ); regularization weight $\lambda_{r}$; anomaly threshold *h*.  2:**Output:** Final global model $\Theta_{g}^{*}$ and anomaly labels $\hat{y}$.  3:**for** each round r=1 to $R_{\max}$ **do**  4:    **Parameter Broadcast:** Server distributes $\Theta_{g}^{\left(r\right)}$ to all clients;  5:    **for** each client k∈{1,…,K} **in parallel do**  6:        Initialize local model $\Theta_{k}^{\left(r\right)}\leftarrow \Theta_{g}^{\left(r\right)}$;  7:        **Local Training:** Train $\Theta_{k}^{\left(r\right)}$ for *E* epochs using Algorithm 1 (Training Phase);  8:        Compute local update: $\Delta_{\Theta_{k}}=\Theta_{k}^{\left(r\right)}-\Theta_{g}^{\left(r\right)}$;  9:        Apply differential privacy: ${\tilde{\Delta }}_{\Theta_{k}}=P\left(\Delta_{\Theta_{k}}\right)+N\left(0,\sigma^{2}I\right)$;10:        Upload ${\tilde{\Delta }}_{\Theta_{k}}$ to server;11:    **end for**12:    **Secure Aggregation:** $\Delta_{\Theta_{g}}^{\left(r\right)}=\sum_{k=1}^{K}\frac{T_{k}}{T_{\mathrm{total}}}{\tilde{\Delta }}_{\Theta_{k}}$;13:    **Update:** $\Theta_{g}^{\left(r+1\right)}=\Theta_{g}^{\left(r\right)}+\Delta_{\Theta_{g}}^{\left(r\right)}$;14:**end for**15:**Anomaly Scoring:** Compute $AD_{\mathrm{score}}\left(X^{\prime }\right)$ using Algorithm 1 (Inference Phase);16:**Threshold Judgment:** $\hat{y}\leftarrow I\left(AD_{\mathrm{score}}\left(X^{\prime }\right)\geq h\right)$;17:**return** $\Theta_{g}^{*},\hat{y}$

In each communication round, the server first broadcasts the current global model parameters $\Theta_{g}^{\left(r\right)}$ to all clients. Then, client *k* performs local training over *E* epochs on its private dataset $D_{k}$, obtaining an updated model and the corresponding parameter difference $\Delta_{\Theta_{k}}^{\left(r\right)}$. To protect sensitive information, a privacy-preserving operator P(·) which combines regularization and norm clipping is applied to the update, followed by Gaussian noise injection to ensure (ε,δ)-differential privacy. The resulting noisy update ${\tilde{\Delta }}_{\Theta_{k}}^{\left(r\right)}$ is transmitted to the server.

In the aggregation phase, the server performs weighted averaging over all received updates, producing the aggregated update $\Delta_{\Theta_{g}}^{\left(r\right)}$ based on client data sizes. The global model is then updated as $\Theta_{g}^{\left(r+1\right)}=\Theta_{g}^{\left(r\right)}+\Delta_{\Theta_{g}}^{\left(r\right)}$ and redistributed to all clients. This iterative process continues until the global model converges.

After convergence, the optimized global parameters $\Theta_{g}^{*}=\left(\eta_{g}^{*},\omega_{g}^{*},\theta_{g}^{*}\right)$ are used to compute anomaly scores for test sequences, following the same multi-branch scoring strategy as in SW-TSAD. This federated framework ensures that the anomaly detection model benefits from diverse client data while rigorously preserving data privacy throughout the training process.

## 4. Results and Discussion

This section presents a comprehensive empirical evaluation of FedSW-TSAD on multivariate time series anomaly detection tasks. It is divided into four parts: (1) a description of the benchmark datasets, (2) a formal statement of the evaluation metrics and implementation guidelines, (3) an overview of the baseline models, and (4) a presentation and analysis of the comparison results with the baselines. Through this structured validation approach, the performance of the proposed model is empirically examined, and its competitive advantages are evaluated relative to existing methods.

### 4.1. Datasets and Experiment Setup

The evaluation involves four publicly available multivariate time series datasets, whose characteristics are summarized in Table 1. The first two datasets, Server Machine Dataset (SMD) [40] and Pool Server Metrics (PSM) [41], are collected from distributed sensing infrastructures in large-scale computing systems. Specifically, SMD includes real-time sensor readings from 28 monitored server nodes, covering performance indicators such as CPU usage and memory activity, with anomalies labeled based on operational event reports. Proposed by eBay, PSM comprises 26-dimensional system-level measurements recorded by monitoring sensors embedded in application servers. Additionally, two sensor-based satellite telemetry datasets from NASA are included: Soil Moisture Active Passive (SMAP) and the Mars Science Laboratory (MSL) [22]. These datasets provide remote sensing data for Earth and planetary monitoring.

To rigorously evaluate FedSW-TSAD’s performance in realistic non-IID federated environments, we carefully designed our data partitioning strategy for each of the four real-world sensor datasets (PSM, MSL, SMD, and SMAP). For all experiments, we simulated five federated clients. The raw time series data from each dataset were divided into distinct, non-overlapping chronological segments. Each of these equally sized temporal segments was then assigned to a unique client. Overall, this time-based partitioning approach is a highly relevant method for inducing non-IIDness in time series federated learning because it reflects the inherent temporal dynamics of real-world systems.

In all experiments, time series data are segmented using a sliding window approach with a window length S=7, where the first $t_{0}=6$ steps serve as the conditioning input and the last τ=1 step is used for prediction. The stride is set to 1, resulting in N=T−S+1 subsequences per series. Min-max normalization is applied to ensure consistency across features:

(21) $$\tilde{x}=\frac{x-\min(X_{\mathrm{train}})}{\max\left(X_{\mathrm{train}}\right)-\min\left(X_{\mathrm{train}}\right)}$$

where $\min(X_{\mathrm{train}})$ and $\max(X_{\mathrm{train}})$ denote the minimum and maximum values across all the training samples, respectively.

Model implementation is based on the PyTorch 1.12.1 framework, with experiments conducted on a single NVIDIA RTX 3090 GPU. The SW-TSAD model is trained until convergence on each dataset in the centralized setting. For federated learning, FedSW-TSAD is deployed with K=5 clients. Each client performs E=5 local training epochs per communication round, with a total of $R_{\max}=50$ global rounds. The batch size is set to m=1024, and model optimization is performed using the Adam optimizer with the learning rate $\phi =1\times 10^{-4}$ and momentum parameters $\left(\psi_{1},\psi_{2}\right)=\left(0.5,0.999\right)$. To ensure privacy, we apply FedSW-TSAD with clipping and Gaussian noise. The privacy budget (ϵ,δ) is set to $\left(2,10^{-5}\right)$, which represents a widely accepted level of differential privacy in practice. During inference, the final anomaly score $AD_{score}$ is computed by combining reconstruction, discrimination, and prediction components with empirically tuned weights: α=0.35, β=0.15, and γ=0.5.

### 4.2. Evaluation Metrics

Anomaly detection performance is assessed using precision (Pre), recall (Rec), and F1-score (F1), which are standard metrics for binary classification in imbalanced settings. In particular, the F1-score, as the harmonic mean of precision and recall, is especially suitable for time-series anomaly detection tasks, where datasets are typically highly imbalanced—i.e., normal instances significantly outnumber anomalous ones. In such cases, the F1-score provides a balanced measure by accounting for both false positives and false negatives, offering a more comprehensive evaluation of detection performance than accuracy alone.

The F1-score is computed based on a tunable threshold applied to the anomaly scores. To estimate the performance upper bound, we follow the evaluation protocol in Su et al. [40] and conduct an exhaustive search over threshold values ranging from $1\times 10^{-4}$ to 1, with 150 evenly spaced points. The best achievable score under this search is reported as F1-best, reflecting the model’s maximum potential under ideal threshold calibration. Additionally, a window-based evaluation criterion is adopted, where a window is considered anomalous if at least one of its constituent time points is identified as anomalous.

### 4.3. Baselines

To validate the effectiveness of the proposed model across different deployment settings, baseline methods are categorized into non-federated (centralized) and federated learning-based approaches. This division enables a clear comparison between SW-TSAD and existing centralized methods, as well as between FedSW-TSAD and existing federated frameworks.

**Non-federated baselines.** This group includes traditional machine learning algorithms, deep generative models, and Transformer-based detectors commonly used in centralized anomaly detection pipelines. LOF [13] detects anomalies by evaluating the local density deviation of a point relative to its neighbors, offering robustness in high-dimensional, unsupervised settings. iForest [14] isolates anomalies via recursive partitioning of the feature space, enabling efficient and accurate detection without probabilistic assumptions. MADGAN [32], a GAN-based framework for multivariate time-series anomaly detection, models complex dependencies through adversarial learning and employs MLP modules to reduce computational cost. USAD [42] enhances anomaly detection by combining adversarial training with an encoder-decoder structure and a signal amplification mechanism. OmniAnomaly [40] incorporates temporal dependencies via stochastic variable modeling, achieving strong performance on real-world sensor time-series benchmarks. Autoformer [43] integrates series decomposition and autocorrelation mechanisms into a deep architecture to improve temporal pattern extraction and forecasting accuracy. Informer [44] introduces the ProbSparse self-attention and a generative decoder, significantly boosting efficiency in long-sequence modeling. FEDformer [45] combines Fourier-based decomposition with Transformer structures to enhance global pattern extraction and scalability. AT [46] proposes a Transformer-based anomaly detector leveraging anomaly-attention and discrepancy-aware learning to capture multi-scale dependencies and distinguish anomalous behaviors. FPT [47] adapts frozen pretrained Transformers from NLP and vision domains for time-series tasks, maintaining model structure while achieving competitive performance and offering theoretical insights through its connection to PCA.

**Federated baselines.** This group consists of models designed for or adapted to the federated setting, where training occurs across decentralized clients. Several Transformer-based models are extended to the federated setting via the FedAvg algorithm [7], including Autoformer(fl), Informer(fl), FEDformer(fl), AT(fl), and FPT(fl). In addition, DeepSVDD [36], a one-class classification method evaluated in the FedTADBench benchmark, and FedAnomaly [37], a variational autoencoder-based distributed detection framework, are included as federated baseline models.

### 4.4. Main Results

Table 2 and Table 3 present the evaluation results comparing the proposed models and baselines under centralized and federated settings across four benchmark datasets (PSM, SMAP, MSL, and SMD). In each setting, the best F1-score is marked in bold, and the second-best is underlined for clarity.

In the centralized setting, SW-TSAD achieves consistently strong results, outperforming all baselines on every dataset. For example, it reaches an F1-score of 97.95% on PSM, exceeding the next-best model FPT by a small margin of 0.88%. On SMAP, it achieves 80.81%, outperforming the best-performing traditional baseline iForest by 10.4 percentage points. This balance of high recall and high precision is also evident in the MSL and SMD datasets, where SW-TSAD achieves F1-scores of 88.35% and 93.50%, respectively, exceeding the best existing models by 3.71% and 1.48%, respectively. Although the margins over strong baselines like FPT, AT, and OmniAnomaly are small, they demonstrate that integrating reconstruction and prediction modules contributes to a more robust and generalizable detector.

The effectiveness of the model is further validated in the federated learning framework. Under a five-client setting, FedSW-TSAD maintains an F1-score of 97.91% on the PSM dataset, with only a 0.04% performance gap compared to centralized training (97.95%). This significantly outperforms other federated learning methods, with an F1-score improvement of 25.55 percentage points over the best-performing alternative, DeepSVDD (72.36%). In the SMAP dataset, the proposed method sustains 100% recall in the federated setting, achieving an F1-score of 79.92%, which is 48.9% higher than the centralized baseline, FedAnomaly (53.66%).

These results highlight the key strength of the proposed framework: while SW-TSAD shows moderate advantages, FedSW-TSAD demonstrates substantial improvement over alternatives. This improvement stems from two core components. First, combining prediction and reconstruction modules enhances anomaly coverage across diverse behaviors. Second, the Sobolev–Wasserstein constraint stabilizes GAN training, while L2-norm clipping regularizes all local modules, mitigating client inconsistency in federated updates. Together, these designs enable FedSW-TSAD to approach centralized performance while preserving privacy. This makes it well-suited for anomaly detection in distributed sensor networks and privacy-sensitive industrial IoT applications, where local heterogeneity and data protection requirements hinder the use of centralized methods.

## 5. Comprehensive Model Analysis

This section presents a comprehensive evaluation of FedSW-TSAD from multiple perspectives, including effectiveness, efficiency, robustness, and privacy. The analysis covers ablation study, system performance, client instability, hyperparameter sensitivity, and the impact of differential privacy mechanisms, providing a well-rounded understanding of the model’s practical behavior in federated anomaly detection scenarios.

### 5.1. Ablation Study

To assess the impact of individual components on model performance, an ablation study was conducted by systematically removing key elements from the FedSW-TSAD architecture. The evaluation focused on the effects of excluding the Temporal Convolutional Network, differential privacy (DP), Sobolev–Wasserstein Constraint (SW Constraint), and Hybrid Anomaly Scoring. The experimental results obtained using four benchmark datasets are presented in Table 4.

1. **The effect of TCN:** Removing the Temporal Convolutional Network module (denoted as w/o TCN) significantly reduces the F1-score across all datasets. For instance, in the PSM dataset, the F1-score decreases from 97.91 to 93.30, while in the MSL dataset, it drops from 86.49 to 80.02. This confirms the importance of the TCN in modeling temporal dependencies essential for accurate anomaly detection.
2. **The effect of DP:** The removal of DP (w/o DP) leads to a slight improvement in the F1-score on certain datasets, such as PSM (from 97.91 to 98.31) and MSL (from 86.49 to 89.24). However, DP remains essential for privacy preservation in federated learning. These findings underscore the trade-off between privacy and performance, which must be carefully managed.
3. **The effect of the Sobolev–Wasserstein constraint:** Excluding the Sobolev–Wasserstein constraint (w/o SW constraint) results in a notable performance decline, particularly in the PSM dataset, where the F1-score drops from 97.91 to 94.01. This suggests that the SW constraint is crucial in enhancing feature alignment across clients, thereby improving overall model robustness in federated environments.
4. **The effect of Hybrid Anomaly Scoring:** To evaluate the contribution of the hybrid scoring mechanism, additional ablation settings were introduced during the inference stage. In the first variant, the prediction-based score $P_{\mathrm{score}}$ was removed, resulting in a reconstruction-only detector denoted as (w/o Prediction Score). In the second variant, the reconstruction-based scores $R_{\mathrm{score}}$ and $D_{\mathrm{score}}$ were removed, simulating a purely prediction-based scheme, denoted as (w/o SWGAN Score). The results exhibit distinct trends across datasets with different anomaly types. On MSL and SMAP, where collective anomalies dominate, the absence of reconstruction-based scores led to significant F1-score drops of 18.57 and 26.32 percentage points. In contrast, excluding the prediction score resulted in relatively minor performance degradation (3.01 and 5.20 points). This suggests that reconstruction-based detection is more effective at capturing structured anomalies affecting multiple dimensions or time steps. Conversely, on PSM and SMD—datasets characterized by point anomalies and abrupt shifts—the prediction score played a more critical role. Removing $P_{\mathrm{score}}$ decreased the F1-score by 18.75 and 30.51 percentage points, respectively, whereas removing reconstruction-based scores led to smaller declines (6.68 and 7.62 points). These findings highlight the complementary nature of the two scoring paradigms: while each offers benefits for specific anomaly types, their combination yields robust and consistently high detection performance across diverse scenarios.

The ablation study demonstrates that the TCN and the Sobolev–Wasserstein constraint are crucial for enhancing detection accuracy, while differential privacy introduces a moderate trade-off between utility and privacy (see Section 5.7). Additionally, the hybrid scoring mechanism significantly improves robustness: removing either the prediction-based or reconstruction-based score leads to substantial performance drops on datasets dominated by different anomaly types. These results highlight the necessity of each component and the importance of combining complementary signals in federated anomaly detection.

### 5.2. System Efficiency Analysis

**Figure 5:**
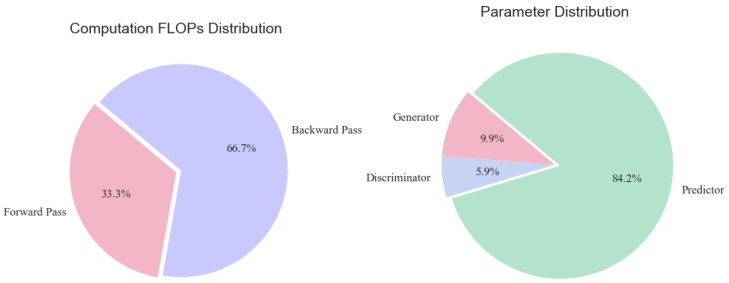
FLOPs and parameter analysis of the proposed model on the MSL dataset. The predictor dominates in terms of both computation and parameter count.

To assess the computational overhead and potential for edge deployment, we conducted targeted experiments on the MSL dataset. Compared to the other datasets used in this study, MSL offers a moderate scale in both training and test set sizes and features the highest input dimensionality (55 sensors), making it a representative choice for evaluating runtime and memory efficiency under realistic multivariate input conditions. As shown in Table 5 and Figure 5, the predictor contains the majority of parameters—189.6K, representing over 84% of the model size. The total floating point operations (FLOPs) per round amount to 302.4 MFLOPs, which remains lightweight given the model’s modular architecture.

**Figure 6:**
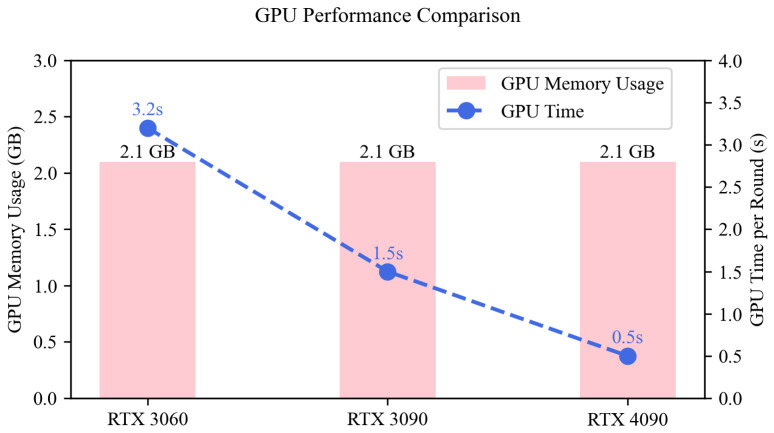
Visualization of runtime performance of the model across different GPU platforms on the MSL dataset.

We further measured the runtime across three GPU platforms of varying capacity (Table 6 and Figure 6). Even with full-precision FP32 operations, the model achieved rapid execution: 3.2 s per round on an RTX 3060 and just 0.5 s on an RTX 4090, with stable memory usage at 2.1 GB across all devices. These figures suggest good scalability and suitability for edge GPUs.

**Figure 7:**
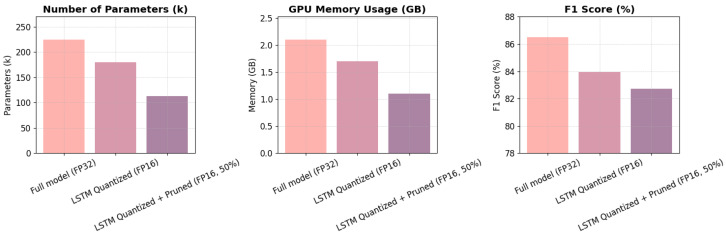
Visualization of the impact of quantization and pruning on model performance and resource consumption, based on the MSL dataset. Quantization and pruning significantly reduce resource requirements.

Given the predictor’s parameter dominance, we applied two efficiency optimizations: 16-bit floating-point quantization and structured pruning at 50% sparsity (Table 7 and Figure 7). Quantization reduced the model size to 180K parameters and memory usage to 1.7 GB, with only a modest F1 decline (from 86.49% to 83.95%). Pruning further compressed the model to 113K parameters and 1.1 GB of memory while retaining 82.71% F1 accuracy. These results indicate that the model can be significantly compressed with minimal performance trade-offs.

Overall, the experiments confirm that the proposed model—despite its use of SWGAN, TCN, and LSTM modules—can maintain low computational costs. The applied compression techniques enable faster inference and reduced memory footprint, supporting deployment on resource-limited edge devices.

### 5.3. Sensitivity to the Number of Clients

The influence of client quantity on model effectiveness was examined by comparing four configurations: a centralized setup (all data on a single server) and federated settings with 5, 10, and 15 clients. Performance was evaluated using the precision, recall, and F1-score across all four datasets. The results are summarized in Table 8.

Overall, the centralized configuration yields the highest performance, with a gradual decline observed as the number of clients increases. On the PSM dataset, for instance, the F1-score drops from 97.95 (centralized) to 97.91 (5 clients), 94.97 (10 clients), and 92.12 (15 clients). A similar trend is observed across other datasets: on SMAP, the F1-score drops from 80.81 to 75.20; on MSL, from 88.35 to 81.38; and on SMD, from 93.50 to 87.66, as the number of clients increases from 1 (centralized) to 15.

This degradation reflects a key trade-off in federated learning: more clients offer improved data locality and privacy but introduce greater statistical heterogeneity and reduce the local data volume, thereby weakening generalization. Across the four datasets, the average F1-score decline from centralized to 15-client configurations ranges from approximately 5.61% (SMD) to 6.97% (MSL), clearly illustrating this trend. These observations underscore the importance of selecting an appropriate client scale to balance performance and deployment constraints in real-world scenarios.

### 5.4. Robustness Evaluation

To evaluate the robustness of the proposed FedSW-TSAD model, we conducted experiments focusing on two key aspects: the model’s ability to handle noisy or corrupted client data and its tolerance to client dropout and intermittent communication during federated training. All robustness experiments were conducted on the SMD dataset, due to its large size and low anomaly rate, which makes it a suitable testbed for evaluating performance under partial corruption or instability. The federated configuration used in these experiments consists of five clients, each trained with non-overlapping partitions of the SMD training data.

In real-world scenarios, client devices may generate noisy or partially corrupted time-series data due to sensor faults or environmental interference. To simulate local data corruption, we randomly selected 20% of clients and added zero-mean Gaussian noise to all training samples on those clients. The noise intensity is controlled via the standard deviation σ of the Gaussian distribution, set to 10%, 15%, or 20% of the average magnitude of the input features. The test data remained clean to assess generalization robustness. Table 9 presents the F1-scores under different noise levels.

As shown in Table 9, the model maintains a relatively high detection performance under mild noise (10–15%), with a decrease in F1-score of less than 7%. Even with 20% noise, the model achieves an F1-score of 80.08%, indicating strong robustness to moderate data corruption. This resilience is attributed to the regularized training of the Sobolev–Wasserstein GAN and the federated aggregation mechanism, which collectively mitigate the influence of outliers.

Federated learning systems often suffer from client unavailability due to network instability or device power constraints. To simulate client unavailability, we apply random dropout during each communication round: each client has a fixed probability (10–40%) of becoming temporarily unavailable and skipping that round. This probabilistic dropout model reflects realistic network instability or device constraints in federated environments. Table 10 provides a summary of the model’s performance under different dropout rates.

The results demonstrate that FedSW-TSAD is tolerant to a moderate level of client dropout. With up to 20% dropout, the F1-score remains above 90%, suggesting minimal degradation. Although performance declines more noticeably beyond 30% dropout, the model still preserves acceptable anomaly detection capability, highlighting its robustness in dynamic federated environments.

### 5.5. Hyperparameter Sensitivity Analysis

In this study, we conducted a hyperparameter sensitivity analysis to examine how the weights of different loss components influence model performance. We focused on three coefficients: α (discrimination loss), β (reconstruction loss), and γ (prediction loss), and designed two rounds of systematic experiments to explore their impact and determine an effective configuration.

**Figure 8:**
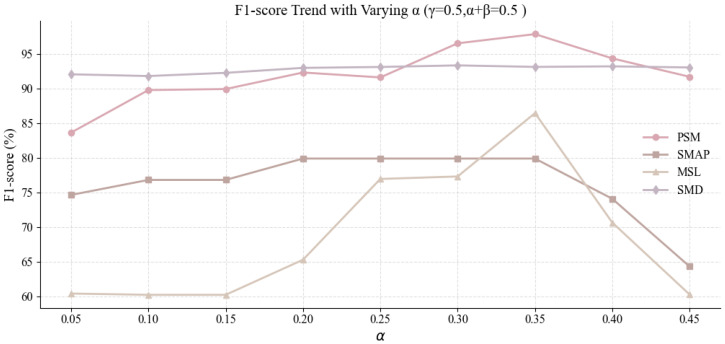
Visualization of model performance as a function of α and β with fixed γ=0.5. The plots reveal a clear upward trend in F1-score with larger α values, supporting the importance of emphasizing the discrimination loss component.

In the first round, we fixed γ=0.5 and maintained α+β=0.5, varying the ratio of α to β to investigate how adjusting the weights of the discrimination and reconstruction components affects performance. The results, summarized in Table 11 and visualized in Figure 8, show a consistent improvement in F1-score with increasing α across the four datasets (PSM, SMAP, MSL, and SMD), peaking when α=0.35 and β=0.15. This configuration yielded F1-scores of 97.91% on PSM, 79.92% on SMAP, 93.17% on SMD, and 86.49% on MSL, reflecting both generalization and robustness. These outcomes indicate that giving more weight to the discrimination term (α) improves anomaly detection. In contrast, reducing the reconstruction term (β) does not noticeably impair performance and may help the model focus on learning more discriminative features.

**Figure 9:**
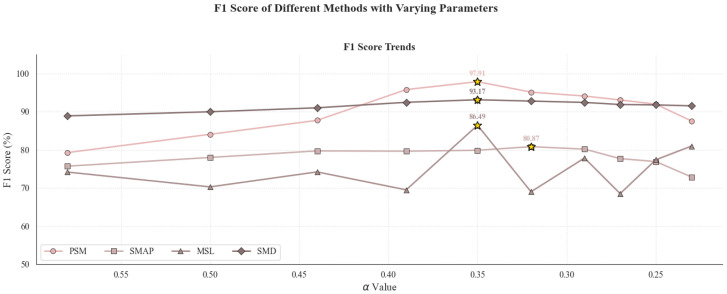
Impact of varying γ on F1-score while holding α:β=7:3 constant. The curves indicate that the model reaches optimal stability and accuracy around γ=0.50, with lower or higher values leading to slight performance degradation.

Guided by these results, we fixed the ratio α:β=7:3 and conducted a second round of experiments to assess how varying γ affects performance, as shown in Table 12 and visualized in Figure 9. We gradually increased γ from 0.17 to 0.67 to test different contribution levels from the prediction term. As γ increased moderately, F1-scores improved across all datasets, with optimal results at γ=0.50. Specifically, the configuration achieved 97.91% on PSM, 79.92% on SMAP, 86.49% on MSL, and 93.17% on SMD, maintaining the performance level from the first round. This consistency supports the effectiveness and stability of assigning a weight of 0.50 to the prediction loss. These findings emphasize the importance of a balanced design among the three loss terms, as both overly low and high values of γ can destabilize performance.

Taken together, the two rounds of sensitivity analysis suggest that the configuration α:β:γ=0.35:0.15:0.50 offers a reliable trade-off among the three loss components, yielding stable and competitive results across various benchmark datasets and providing practical guidance for model tuning.

### 5.6. Privacy-Utility Trade-Off

To assess the practical impact of differential privacy on TSAD performance, we conduct a systematic evaluation of the trade-off between privacy protection and model utility. While previous experiments confirm that introducing DP noise may degrade performance, this subsection quantitatively analyzes how varying privacy budgets affect results under the (ϵ,δ)-DP framework.

Specifically, we fix $\delta =10^{-5}$ and vary the privacy budget ϵ∈{1,2,3,4}. For each setting, we calculate the corresponding noise multiplier σ and retrain the FedSW-TSAD model with identical optimization parameters. An additional baseline with ϵ=∞ represents the non-private case. Precision, Recall, and F1-score are evaluated across four benchmark datasets: PSM, SMAP, MSL, and SMD.

As shown in Table 13, model performance improves consistently as ϵ increases, which corresponds to reduced DP noise and weaker privacy guarantees. For example, with ϵ=1, the average F1-score is only 85.79, reflecting a noticeable degradation in utility. When the privacy budget is relaxed to ϵ=4, the average F1 rises to 91.25, approaching the non-private baseline of 91.69.

### 5.7. Case Study: Impact of Differential Privacy on Federated Learning

**Figure 10:**
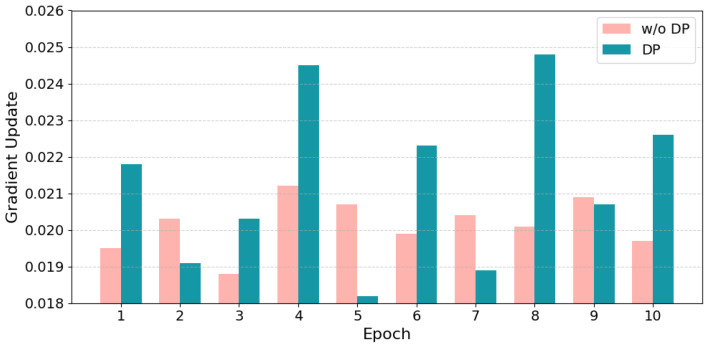
Visualization of gradient update ablation experiment Differential Privacy (DP) vs w/o DP.

To evaluate how differential privacy influences the training dynamics of FedSW-TSAD, gradient updates were recorded over 10 consecutive epochs on the SMAP dataset with five federated clients. Figure 10 illustrates the dynamic evolution of gradient updates between the non-private setting (w/o DP) and the DP-protected scenario using a grouped bar chart.

To determine whether the observed difference is statistically significant, a two-sample *t*-test was applied. For two equally sized groups ($n_{1}=n_{2}=10$), the degrees of freedom were computed as:

The statistical significance of the observed difference was assessed using a two-sample *t*-test. For two equally sized groups ($n_{1}=n_{2}=10$), the degrees of freedom were computed as:

(22) $$n_{1}+n_{2}-2=18$$

The t-statistic was calculated using:

(23) $$t=\frac{{\bar{X}}_{DP}-{\bar{X}}_{w/o\;\mathrm{DP}}}{\sqrt{\frac{s_{DP}^{2}}{n_{DP}}+\frac{s_{w/o\;\mathrm{DP}}^{2}}{n_{w/o\;\mathrm{DP}}}}}$$

With mean gradient update values ${\bar{X}}_{DP}=2.13\times 10^{-2}$ and ${\bar{X}}_{w/o\;\mathrm{DP}}=2.01\times 10^{-2}$, the analysis yields a *t*-value of 2.37 and a *p*-value of 0.029, indicating that under the null hypothesis (no difference in gradient updates between the two groups), the probability of observing such an extreme result is only 2.9%.

Since *p* < 0.05, the null hypothesis is rejected. To assess the strength of the observed difference, Cohen’s *d* was computed:

(24) $$d=\sqrt{\frac{|{\bar{X}}_{DP}-{\bar{X}}_{w/oDP}|}{\frac{\left(n_{DP}-1\right)s_{DP}^{2}+\left(n_{w/oDP}-1\right)s_{w/oDP}^{2}}{n_{DP}+n_{w/oDP}-2}}}=0.74$$

The resulting effect size suggests a moderate impact of DP-induced noise on local gradients (d > 0.5). These findings demonstrate that while DP introduces meaningful variability, the model remains trainable with acceptable performance, highlighting the feasibility of privacy-preserving training under FedSW-TSAD.

## 6. Conclusions

Distributed sensor networks are increasingly deployed in industrial and environmental systems, where decentralized sensing and strict privacy constraints render centralized anomaly detection approaches impractical. To address these challenges, this study presents FedSW-TSAD, a federated learning framework tailored for time series anomaly detection in such settings. The framework integrates Sobolev–Wasserstein GANs to stabilize generative modeling, temporal convolutional networks for feature extraction, and a hybrid scoring mechanism to improve robustness under heterogeneous anomaly types. These components collectively address two core difficulties in federated TSAD: unstable training and limited generalization across clients.

Comprehensive experiments on four real-world sensor datasets demonstrate that FedSW-TSAD consistently outperforms both centralized and federated baselines. Notably, it achieves near-centralized detection accuracy under federated settings while preserving privacy through L2-norm-constrained differential privacy. These results underscore the significance of designing anomaly detection models that align with the distributed nature of modern sensor networks, offering a scalable and privacy-aware solution for critical applications such as industrial IoT, predictive maintenance, remote diagnostics, and smart healthcare.

While FedSW-TSAD demonstrates strong performance across multiple datasets, several limitations warrant future exploration. First, the framework relies on fixed-size sliding windows for segmenting time series, which may be suboptimal for sequences with dynamic temporal patterns. Second, although the model simulates concept drift through time-based data partitioning, it lacks real-time adaptability to long-term distributional changes. Addressing these challenges, future work may incorporate adaptive windowing strategies based on signal characteristics and develop online adaptation mechanisms for sustained deployment. Furthermore, explainable anomaly detection techniques—such as attention-based visualizations, perturbation-based saliency analysis, or post hoc feature attribution—could enhance model interpretability and support trustworthy deployment in safety-critical environments.

## Figures and Tables

**Table 1:** Dataset statistics.

Datasets	Train	Test	Dimensions	Anomaly Rate (%)
PSM	132,481	26,497	25	28
MSL	58,317	73,729	55	11
SMAP	135,183	427,617	25	13
SMD	708,405	708,420	38	4

**Table 2:** The main results of comparing SW-TSAD and centralized baseline models are presented. “Pre,” “Rec,” and “F1” represent the precision, recall, and F1-score, respectively, expressed as percentages.

Model	PSM			SMAP			MSL			SMD		
	Pre	Rec	F1	Pre	Rec	F1	Pre	Rec	F1	Pre	Rec	F1
LOF	15.49	100.00	26.83	2.05	100.00	4.01	11.52	100.00	20.67	7.90	100.00	14.65
iforest	19.27	47.93	27.49	54.33	100.00	70.41	79.28	85.93	82.47	77.98	88.60	82.95
OmniAnomaly	75.99	84.96	80.22	50.06	100.00	66.72	69.74	80.28	74.64	87.40	97.16	92.02
USAD	100.00	20.91	34.58	28.37	100.00	44.20	76.77	92.82	84.04	67.95	91.15	77.86
MADGAN	82.04	80.34	81.18	23.05	100.00	37.47	80.78	86.34	83.47	63.05	89.59	74.01
Autoformer	99.94	79.06	88.28	\	\	\	76.93	76.50	76.71	78.45	65.10	71.15
Informer	97.29	80.59	88.15	\	\	\	79.79	74.73	77.18	90.28	75.24	82.08
FEDformer	99.98	81.69	89.91	\	\	\	90.61	69.02	78.35	76.78	59.72	67.19
AT	95.70	95.34	95.52	\	\	\	69.14	86.48	76.85	90.34	82.34	86.16
FPT	98.36	95.82	97.07	\	\	\	81.10	80.35	80.72	87.60	80.79	84.06
SW-TSAD (Centralized)	97.99	97.91	**97.95**	67.80	100.00	**80.81**	88.53	88.18	**88.35**	94.10	92.91	**93.50**

**Table 3:** The main results of comparing FedSW-TSAD and federated baseline models are presented.

Model	PSM			SMAP			MSL			SMD		
	Pre	Rec	F1	Pre	Rec	F1	Pre	Rec	F1	Pre	Rec	F1
Autoformer(fl)	97.77	78.88	86.64	\	\	\	84.09	65.57	72.66	74.92	82.30	77.23
Informer(fl)	77.98	59.58	64.11	\	\	\	80.34	67.90	72.12	77.44	91.18	83.08
FEDformer(fl)	76.69	58.54	62.64	\	\	\	79.16	66.95	71.36	76.64	89.58	81.66
AT(fl)	87.02	83.57	84.63	\	\	\	81.77	69.40	73.93	87.02	83.57	84.63
FPT(fl)	84.93	80.08	81.49	\	\	\	70.90	73.25	71.85	84.93	80.08	81.49
DeepSVDD	96.57	64.41	72.36	\	\	\	77.69	69.37	72.26	86.01	87.02	85.77
FedAnomaly	\	\	\	67.39	44.58	53.66	58.08	58.57	58.33	\	\	\
FedSW-TSAD (5 clients)	98.15	97.68	**97.91**	66.56	100.00	**79.92**	87.14	85.84	**86.49**	93.73	92.61	**93.17**

**Table 4:** Ablation experiments results.

Model	PSM			SMAP			MSL			SMD		
	Pre	Rec	F1	Pre	Rec	F1	Pre	Rec	F1	Pre	Rec	F1
FedSW-TSAD (5 clients)	98.15	97.68	97.91	66.56	100.00	79.92	87.14	85.84	86.49	93.73	92.61	93.17
w/o TCN	95.40	91.28	93.30	60.12	95.09	73.67	79.83	80.21	80.02	88.16	87.33	87.74
w/o DP	98.59	98.04	98.31	71.30	100.00	83.24	92.13	86.52	89.24	94.08	98.37	96.18
w/o SW constraint	93.73	94.30	94.01	63.92	100.00	77.99	85.37	83.87	84.61	92.12	91.17	91.64
w/o Prediction Score	74.10	84.96	79.16	59.65	100.00	74.72	94.56	74.72	83.48	100.00	45.63	62.66
w/o SWGAN Score	92.32	90.16	91.23	44.25	100.00	61.35	43.04	99.95	60.17	81.39	90.16	85.55

**Table 5:** The number of parameters and FLOPs for each model component on the MSL dataset. The predictor accounts for most of the parameters and computational cost.

Component	Number of Parameters
Generator	22,345
Discriminator	13,215
Predictor	189,596
Total	225,156
**Computation Stage**	**FLOPs (MFLOPs)**
Forward pass	100.8
Backward pass	201.6
Total	302.4

**Table 6:** The runtime performance of the model across different GPU platforms on the MSL dataset. The model maintains low memory usage and high execution efficiency.

Metric	Equipment		
	RTX 3060	RTX 3090	RTX 4090
FLOPs per round	302.4 MFLOPs	302.4 MFLOPs	302.4 MFLOPs
GPU time per round	3.2 s	1.5 s	0.5 s
GPU memory usage	2.1 GB	2.1 GB	2.1 GB

**Table 7:** Impact of quantization and pruning on model performance and resource consumption on the MSL dataset. The model remains effective even with significant parameter reduction.

Configuration	F1 Score (%)	GPU Memory (GB)	Parameters (k)
Full model (FP32)	86.49	2.1	225
LSTM Quantized (FP16)	83.95	1.7	180
LSTM Quantized + Pruned (FP16, 50%)	82.71	1.1	113

**Table 8:** The results of federated learning performance across different numbers of simulated clients.

Different Numbers of Client	PSM			SMAP			MSL			SMD		
	Pre	Rec	F1	Pre	Rec	F1	Pre	Rec	F1	Pre	Rec	F1
Centralized	97.99	97.91	97.95	67.80	100.00	80.81	88.53	88.18	88.35	94.10	92.91	93.50
5 clients	98.15	97.68	97.91	66.56	100.00	79.92	87.14	85.84	86.49	93.73	92.61	93.17
10 clients	95.21	94.75	94.97	64.56	97.00	77.52	84.53	83.26	83.90	90.92	89.83	90.37
15 clients	92.35	91.91	92.12	62.63	94.09	75.20	81.99	80.77	81.38	88.19	87.14	87.66

**Table 9:** Performance under varying levels of client data noise on the SMD dataset.

Model	Clean Data F1 (%)	Noisy Data F1 (%)	Δ F1 (%)
FedSW-TSAD (10% noisy)	93.17	89.25	3.92
FedSW-TSAD (15% noisy)	93.17	86.31	6.86
FedSW-TSAD (20% noisy)	93.17	80.08	13.09

**Table 10:** Performance under different client dropout rates on the SMD dataset.

Dropout Rate	F1 (%)	Precision (%)	Recall (%)
0% (FedSW-TSAD)	93.17	93.73	92.61
0.10	92.59	93.15	92.04
0.20	90.98	91.85	90.12
0.30	88.45	89.60	87.33
0.40	83.47	85.24	81.77

**Table 11:** F1-scores under varying α and β settings with fixed γ=0.5 on four benchmark datasets. As α increases while β decreases (maintaining α+β=0.5), the F1-scores improve across most datasets. The best F1-score for each dataset is highlighted in bold. Overall, the best configuration is observed at α=0.35, β=0.15.

Fixing γ = 0.5		PSM			SMAP			MSL			SMD		
α	β	Pre	Rec	F1	Pre	Rec	F1	Pre	Rec	F1	Pre	Rec	F1
0.05	0.45	82.33	85.06	83.67	59.56	100.00	74.66	43.27	99.95	60.40	91.75	92.45	92.10
0.10	0.40	95.27	84.96	89.82	62.38	100.00	76.83	43.09	99.95	60.22	91.26	92.45	91.85
0.15	0.35	91.38	88.60	89.97	62.38	100.00	76.83	43.09	99.95	60.22	92.18	92.45	92.31
0.20	0.30	96.45	88.60	92.36	66.56	100.00	**79.92**	48.48	99.95	65.29	93.61	92.45	93.03
0.25	0.25	90.24	93.11	91.65	66.56	100.00	**79.92**	62.58	99.95	76.97	93.89	92.45	93.16
0.30	0.20	97.17	95.99	96.58	66.56	100.00	**79.92**	63.06	99.95	77.33	94.18	92.61	**93.39**
0.35	0.15	98.15	97.68	**97.91**	66.56	100.00	**79.92**	87.14	85.84	**86.49**	93.73	92.61	93.17
0.40	0.10	97.24	91.73	94.40	58.85	100.00	74.10	54.65	99.95	70.66	93.04	93.47	93.25
0.45	0.05	89.53	94.07	91.74	47.39	100.00	64.31	43.13	99.95	60.26	94.05	92.14	93.09

**Table 12:** F1-scores under varying γ values with fixed α:β=7:3 on four benchmark datasets. The best F1-score for each dataset is highlighted in bold. Moderate increases in γ lead to consistent performance gains, with γ=0.50 yielding the most stable and accurate results across datasets.

Fixing α:β=7:3			PSM			SMAP			MSL			SMD		
α	β	γ	Pre	Rec	F1	Pre	Rec	F1	Pre	Rec	F1	Pre	Rec	F1
0.58	0.25	0.17	72.33	87.79	79.31	60.96	100.00	75.75	61.17	94.30	74.21	86.48	91.54	88.94
0.50	0.21	0.29	85.27	82.99	84.11	63.99	100.00	78.04	54.23	99.95	70.32	87.58	92.61	90.02
0.44	0.19	0.38	91.38	84.44	87.77	66.34	100.00	79.76	65.42	85.84	74.25	89.56	92.61	91.06
0.39	0.17	0.44	98.73	93.11	95.84	66.23	100.00	79.68	53.33	99.95	69.55	92.39	92.61	92.50
0.35	0.15	0.50	98.15	97.68	**97.91**	66.56	100.00	79.92	87.14	85.84	**86.49**	93.73	92.61	**93.17**
0.32	0.14	0.55	91.97	98.59	95.17	67.88	100.00	**80.87**	52.74	99.95	69.04	92.44	93.21	92.82
0.29	0.12	0.58	94.68	93.60	94.14	66.99	100.00	80.23	63.81	99.95	77.89	91.75	93.21	92.47
0.27	0.12	0.62	92.64	93.60	93.12	63.57	100.00	77.73	52.17	99.95	68.56	90.43	93.47	91.92
0.25	0.11	0.64	90.49	93.60	92.02	62.55	100.00	76.96	63.19	99.95	77.43	91.76	91.88	91.82
0.23	0.10	0.67	87.17	88.05	87.61	57.38	100.00	72.92	71.02	94.30	81.02	91.26	91.88	91.57

**Table 13:** F1-scores of FedSW-TSAD under varying privacy budgets ϵ, evaluated on four benchmark datasets. A larger ϵ implies weaker privacy but higher utility. The baseline ϵ=∞ represents the non-private setting.

Privacy Budget ϵ	PSM			SMAP			MSL			SMD			Avg.F1
	Pre	Rec	F1	Pre	Rec	F1	Pre	Rec	F1	Pre	Rec	F1	
ϵ=1	93.72	95.14	94.42	60.65	100.00	75.51	82.03	85.55	83.75	91.11	87.90	89.48	85.79
ϵ=2	98.15	97.68	97.91	66.56	100.00	79.92	87.14	85.84	86.49	93.73	92.61	93.17	89.37
ϵ=3	98.15	97.88	98.01	68.31	100.00	81.17	88.89	86.23	87.54	94.00	94.42	94.21	90.23
ϵ=4	98.35	98.00	98.17	70.98	100.00	83.03	90.47	86.23	88.30	94.00	97.07	95.51	91.25
ϵ=∞	98.50	98.00	98.23	71.30	100.00	83.24	92.00	86.50	89.16	94.00	98.37	96.13	91.69

## Data Availability

Publicly available datasets were analyzed in this study. These datasets include the Server Machine Dataset (SMD) [40], Pool Server Metrics (PSM) [41], Soil Moisture Active Passive (SMAP) [22], and Mars Science Laboratory (MSL) [22].
